# Correlation between adequate nursing staff and the hospital performance: Case Study in Tehran University of Medical Sciences Hospitals

**Published:** 2015

**Authors:** S Azari, S Mokhtari, A Aliyari, M Mohammadi, M Afroozi, M Salimi, GH Azari

**Affiliations:** * Health Management and Economics Research Center, Iran University of Medical Sciences, Tehran, Iran,; ** School of Public Health, Tehran University of Medical Sciences, Tehran, Iran,; *** School of Public Health, Tehran University of Medical Sciences, Tehran, Iran,; **** Farabi Hospital, Tehran University of Medical Sciences, Tehran, Iran,; ***** Student Research Committee, Kermanshah University of Medical Sciences, Kermanshah, Iran,; ****** Health Services Management Research Center, Institute for Futures Studies in Health, Kerman University of Medical Sciences, Kerman, Iran,; ******* Department of Health Service Management, Islamic Azad University-Science and Research Branch, Tehran, Iran

**Keywords:** estimation, nursing staff, hospital, personnel standards, Pabon Lasso

## Abstract

**Background:** As the biggest collection among various collections of hospital employees, nurses perform a vital role in the progress of the hospital actions and the improvement of community health.

**Objectives:** This research intended to find the relationship between the shortage of nursing workforce and hospitals performance of Tehran’s Medical Sciences University.

**Materials and Methods:** The current analysis was a cross-sectional definitive research, which was conducted in selected educational hospices associated to Tehran University of Medical Sciences during the year 2010. While using three researcher-made questionnaires, information was obtained from all clinical, paraclinical, commercial, managerial and support units of hospitals. Information was investigated according to the patterns of Iran's Healthcare Ministry and Medical Education by employing Excel software. Pabon Lasso model was used for performance measurement during the year 2010. The mixture of 3 indicators was applied to determine the period of stay, bed occupancy time and bed turnover.

**Results:** The outcomes revealed that the personnel of nursing, in the 18 hospital wards (66.67%) were at levels below the standards, just one ward (3.7%) reached the standards requirements, and the remainder of the hospital wards (29.62%) were at greater levels than the standard requirements. Both hospitals were near the value 4 in the Pabon Lasso model. The correlation analysis among the nursing shortage and performance showed a meaningful relationship (P<0.05).

**Conclusion:** Frequently, the examined hospices were challenged with a shortage of nursing workforce and the dissemination of workforce was not suitable. A decent supervision of workforce in conformity with the shortage of staff compensated and achieved the rules needed for the hospital’s nursing workforce and this would guide to an improvement in the performance of the hospitals’ actions and could present gratification for the personnel.

## Introduction

Attitudes toward the workforce and personnel resources have changed simultaneously with the wonderful changes we witness in today’s word and also along with the information blast, globalization, and similar cases, as they were considered the main factors that cause these changes. At present, none of the managers has an instrumental attitude toward human resources, employees are valuable assets of an association, and so many scholars have been trying to find an effective method to create and keep these assets [**1**]. The Nobel prize winner, Simon Kuznets, reasoned that a true asset of most developed countries is not the fact that they have entrance to great physical and material assets but the collection of concentrated knowledge which is a result of experimental knowledge and discoveries resulted in this experimental science and knowledge, of course, the capacity and presented educations are also considered important factors in the effective application of this science [**2**]. Undoubtedly, there were hospital criteria and standards applied along with the proper management, which directed to the efficiency and performance of the hospital services. Meanwhile, the workforce is the primary and most significant section, creating the hospital as an organization. The significance of the workforce in presenting hospital services is undeniable and without a proper and educated workforce, hospital activities will be deranged. In fact, a decent arrangement of doctors with needed specialties, nurses, technicians, nurse assistants, have a significant and vital role in the proper working routine of the hospital and the presentation of hospital services [**3**]. The various models and methods were presented to predict the workforce at the universal level and to be based on various time periods. Goodman and Viant have presented the effective factors on developing these methods and models and also the application limits of each one on the long term planning of workforce during the past 100 years [**4**]. The workforce was considered one of the most valuable resources and assets of the hospital and its shortage or surplus could affect the quality of services provided to the patients. Most problems in hospitals were the result of the lack in the workforce or improper arrangement of the workforce [**5**,**6**]. Based on the results presented in a research by Arab & et al. during 2010, studied hospitals faced a workforce shortage and did not have a right supervision and preparation of workforce. Also, there were various investigations on the nurses’ dissatisfaction with the hospital, which was mentioned, and its major reason was the shortage in the amount of nurses [**7**,**8**]. It was essential to explain that the medical staff made up more than 70 percent of the hospital’s workforce and, considering the current expenses of the hospital, they devoted 65 to 70 percent of these expenses to themselves. The most important issue was that based on the reports of the Healthcare Ministry, the bed occupancy coefficient in Iran’s hospitals not exceeding around 60, just about the active beds [**9**]. The final aim of the human resources activities was to provide a proper number of competence employees to satisfy inpatients in hospitals. Hospitals required a certain number of competent individuals to ensure it would perform its purpose and satisfy patients’ needs. In this sense, the workforce was determined by considering the personnel’s working volume. This method used the determined working volume for the hospital’s personnel. This index included the number of inpatients divided on the number of surgeries, number of births, number of inpatients, the number of outpatient clinics, personnel education, visit in the house, etc. For each of mentioned indexes, standard activities were defined. These standards were represented by the rate of time spent for each of these actions [**10**]. Planning the workforce predicted the organizations’ future supply and regular demanding for employees. By guaranteeing the number and type of required employees, the human resource unit can better predict absorption, selection, education, career planning and other activities. If the organization is not supplied by a proper number and type of workforce, the planning might fail. Executive managers recognized that the main success key in planning is the human resource because competent employees ease the successful execution of plans [**11**]. The main goal of the present research was to estimate the required workforce of hospitals based on the pattern suggested by the Healthcare Ministry. The present research was a step toward adjusting with the Hospitals’ personnel standards and by doing so, lacks and surpluses of required workforce in Tehran Medical University’s hospitals would become clear and would also determine what type of career suffers from these shortages and surpluses and finally it would also be determined the way this information is combined with hospital performance.

## Materials and methods 

The current research is of health system studies type and it was done in descriptive–analytical method. The studied society in the present study includes all sections and units with nursing group personnel (nurse, nurse’s aide, nurse’s aide assistant) in 2 hospitals. Choosing these two hospitals was due to the access to data concerning the nursing level in all wards.

Data were collected by means of researcher built questionnaires that were designed by means of previous studies. The present study used three types of forms to collect data, as it follows: 1 related to authorities of selected clinical condition, the workforce of these sections was ascertained by these means. 2: related to medical records section, which was used to ascertain a percentage of sections of bed occupation, number of operating beds and the median of the patients’ stay in clinical sections and 3 used to ascertain the condition of the current workforce and also to study personnel structure designed by the hospitals’ staff department director. After gathering the needed data, lacks and excesses of nursing workforce of studied hospitals were determined based on career types in various sections of the hospital and the required nursing workforce was evaluated.

In order to evaluate the performance of hospitals, Pabon Lasso model combing 3 indicators was used: length of stay, bed occupancy rate, and bed turnover rate defining which hospitals were in a certain region of efficiency. Pabon Lasso model divided hospital performance into 4 areas: area one - hospitals with low bed occupation rate and bed turnover, area two - hospitals with low bed occupancy rate and high bed turnover, area three - hospitals with high bed occupancy rate and bed turnover and finally, area four - hospitals with high bed occupancy rate and low bed turnover. Due to ethical issues, the name of the hospital was not mentioned. Data were analyzed by means of Excel software, descriptive statistic indexes and standards handbook suggested by the Healthcare Ministry. 

**Findings**

Bed occupation rate, the period of stay and bed turnover of 2 hospitals could be seen in **Table 1**. According to Pabon Lasso model, both hospitals were placed in region 4 of efficiency, which had a large bed occupancy rate and low bed turnover, which showed that these hospitals admitted patients with more complicated conditions and an increment in the ordinary length of stay, which led to an increment in cost and need for more personnel.

**Table 1:** Efficiency region of hospitals according to Bed occupation rate, period of stay and bed turnover

hospital	Bed occupancy rate	the length of stay	bed turnover	Region of efficiency
A	87.11	6.65	32.76	4
B	88.36	6.73	44.13	4

Both examined hospitals were public and educational-medical centers. The total count of the current nursing workforce and required number were determined based on the pattern suggested by the Healthcare Ministry for hospitals and the results were displayed in **Table 2**. 

There were 392 nursing organizational posts in Hospital B. Based on the rules of the Healthcare Ministry, this hospital should have had 794 organizational jobs related to nursing staff. Among 16 sections studied in Hospital B, four sections had a nursing surplus based on the pattern recommended by the Healthcare Ministry and the other suffered a shortage in the nursing workforce. Based on this pattern, the maximum shortage existed in ICU (36) and the minimum shortage existed in the newborns section (1). Generally, the distance or difference of the existing condition from the model suggested by the Healthcare Ministry was of 139 individuals (**Table 2**). 346 working careers existed in Hospital A, which should have had 356 nurses, based on the criteria of the Healthcare Ministry. Among 11 studied sections, 4 had a nursing surplus based on the pattern suggested by the Healthcare Ministry, a section was proper based on the pattern and the rest suffered a staff shortage. Based on the model suggested by the Healthcare Ministry the maximum shortage was observed on the orthopedic section (10) and the minimum shortage was observed in the nursing office which was adjusted based on the model. The largest surplus was also seen in the internal section (-1) and the minimum surplus was observed in the general surgeries (-1). Generally, the existing difference of the present condition from the pattern suggested by the Healthcare Ministry was of 30 individuals (**Table 2**). 

The correlation analysis confirmed that there was a meaningful relationship within the nursing staff shortage and the region of efficiency (r= 0.82, P<0.05).

**Table 2:** Condition of studied hospitals’ workforce based on the model proposed by the Healthcare Ministry

Difference of existing condition with the condition suggested by the Healthcare Ministry	Nurses based on model suggested by the Healthcare Ministry	Existing nursing workforce	Active bed number	Bed occupation coefficient	section	Hospital A	Difference of existing condition with the condition suggested by the Healthcare Ministry	Nurses based on model suggested by the Healthcare Ministry	Existing nursing workforce	Active bed number	Bed occupation coefficient	section	Hospital B
-7	17	24	18	2.76	Kidney and urethra surgery		10	22	12	15	1.75	CCU	
10	38	28	42	2.76	orthopedic		36	97	61	31	8.76	ICU	
-8	13	21	24	2.98	internal		5	20	15	6	2.82	NICU	
4	22	18	27	8.94	Digestive system		1	10	9	8	5.12	Newborns	
1	42	43	27	8.75	infectious		8	33	25	36	7.60	Orthopedic	
1	15	14	18	8.94	lung		16	36	20	17	4.79	Nerves, internal	
6	41	35	23	9.65	CCU		16	36	20	40	4.71	General surgery	
8	57	49	18	2.73	ICU		7	52	45	33	8.80	Emergency	
-1	59	60	66	1.84	General surgery		2	12	10	7	4.59	Kidney transplantation	
-7	36	43	40	1.87	Cardio surgery		4	44	20	30	7.68	Lung	
0	11	11	-	-	Nursing office		-9	74	63	34	3.85	Brain and bone transplantation	
30	351	346	303	-	total		8	23	15	24	7.73	Brain and neurology surgery	
							-4	30	34	24	6.94	Internal hematology	
							26	44	17	44	3.81	Women and birth	
							-2	5	7	7	5.80	Post CCU	
							-7	11	19	-	-	Nursing office	
							139	549	392	356	-	total	

## Discussion and conclusion 

People working in medical careers are considered among the very significant group of workforce performing medical and healthcare services. Unique features of the medical group jobs, such as the effect on health, which is one of the most vital aspects of the mankind’s life, makes it necessary to pay attention to planning to considering the future in this field. Consideration and giving attention to workforce planning in the healthcare area is important from two points of view. First, the workforce is rated one of the essential and basic parts in the efficient service providing; second, a magnificent part of the healthcare section financial resources is used to fund wages of this workforce [**12**]. Now, third world countries devote around 60 to 80 percent of their healthcare and medication share to their hospitals while this percent is around 38 percent for hospitals in advanced countries and the rest is devoted to non-hospital medical and healthcare services. Due to this, it is very important to calculate hospital staff expenses in these countries [**13**]. Based on the results from evaluations in examined hospitals and comparing them with the current condition, we realized that the nursing workforce distribution is not balanced in various sections of the hospital and it does not follow the standards. Among 16 sections of Hospital B, 12 sections had less workforce in comparison to the pattern suggested by the Healthcare Ministry and 4 had a higher level than suggested by the Healthcare Ministry. This is while the workforce distribution in the sections of Hospital A was different in a manner that in comparison to the model suggested by the Healthcare Ministry, 4 sections had less workforce, 6 sections had more workforce and finally, one section was adjusted based on the pattern suggested by the Healthcare Ministry. Studying the condition of nursing workforce in the present research and comparing it with the existing researches indicated a lack of the nursing workforce in the examined hospitals. 

The results presented by Akbari & et al. researches showed that between 92 hospital sections studied in Lorestan Medical University hospitals, only 18 sections were adjusted based on the pattern presented by the Healthcare Ministry and 16 sections were better than the suggested rules and the remainder of 58% of the sample society were far from the existing measures and standards [**3**]. In Mostafaiye’s study about hospitals of Medical University of Tehran, he found out that 85.1% of the sections had a shortage, 5.31% had a surplus, and 9.57% had an exact right count of nurses, based on the criteria of the Healthcare Ministry [**14**]. In the ICUs, responsible for taking care of patients in a sensitive condition both in the hospitals lack of nursing workforce was observed. Abrishamkar also determined that the ICUs faced a lack of the nursing staff and lacked an accurate and precise management of workforce [**15**]. The nursing office was one of the units in which the quality and quantity of the workforce’s number was very important because it was in chrge for monitoring and managing the Hospital confining sections’ activities. In our hospitals studies, no shortage was recognized in the required number of workforce in the nursing office based on the model suggested by the Healthcare Ministry, it was to say that there were 8 surplus employees in the nursing staff of Hospital B in comparison with the pattern presented by the Healthcare Ministry. Hospital A had the exact number of the nursing staff in comparison with the pattern suggested by the Healthcare Ministry and there was no shortage in the nursing office, which could be recognized as one of strength points of this hospital. Results of this research showed that studied hospitals faced a lack of the nursing staff and the highest shortage was related to Hospital B with 139 individual’s shortage. The planning regarding the making up of this personnel shortage and reaching the personnel standard level in all hospital sections and presenting the required educations to every sectors director or manager in relation to proper and correct management and planning of workforce of the parts will increase the performance and effectiveness of hospital actions [**16**]. Results of Bahadori study in Iran also showed that 89.5% of hospital beds encounter with low nursing personnel, which is in accordance with our study [**17**]. Based on statistical reports about hospitals incorporated by the Healthcare Ministry, treatment and medical educations were developed; the bed occupation coefficient in Iran’s hospitals did not pass 60 active beds. It is obvious that this rate is less than 50 percent regarding the hospital’s permanent beds. This way, the enormous expenditure that human resources spends to manage hospitals and other healthcare-medical centers requires a great and increasing attention [**13**]. A comparative study about the workforce index and proportion of its distribution in Hospital A, which was done for the Healthcare Ministry, the treatment and Medical Training by Sedghiani showed the truth that in the developed countries’ hospitals, despite the use of advanced technology, which headed to a reduction in the workforce, based on hospitals condition of being educational, non-educational and singular expert, generally 3 or 4 workforce were considered. 

The results of the current study revealed that there is a meaningful connection between the efficiency area of the hospitals and condition of nursing staff in both hospitals. Both hospitals had a high bed occupancy rate with low bed turnover, which needed more personnel in order to consider the best patient treatment and follow up while none of these hospitals had a good situation regarding the nursing personnel. Based on the results of Mark's study, 60% of the unit of service provision was at a lower efficiency level, which needed a decrease of the hours of working of nurses and this led to an adequate number of the nursing personnel [**18**]. Sovie also implied that the outcomes of the hospitals depend on the nursing working hours, which showed the importance of number and hours of nurse staffs on hospital performance [**19**]. Everhart’s study also confirmed a good relationship within the number of the nursing staffs and the financial performance of hospitals [**20**]. What should be reminded is that this number of the workforce is not determined based on guessing but based on the evaluating workforce and time consumption of services methods. The most significant point is that now, the Healthcare Ministry’s hospitals only hold common standards to determine the number and design the workforce has and the personnel standards of the Healthcare Ministry. Generally, in all hospitals of the country, especially hospitals in which the bed’s capacity was not properly used and the median of hospital bed occupancy was low, such as the examined hospitals, managing the hospitals use of less workforce and usually with improper design was applied, which directed to an irreparable damage to the body of the country’s health system and so many facilities and capacities of the hospitals, so, the great investments on them were left untouched. 
